# Effect of Dietary *ω*-3 Polyunsaturated Fatty Acid DHA on Glycolytic Enzymes and Warburg Phenotypes in Cancer

**DOI:** 10.1155/2015/137097

**Published:** 2015-08-03

**Authors:** Laura Manzi, Lara Costantini, Romina Molinari, Nicolò Merendino

**Affiliations:** ^1^Tuscia University, Department of Ecological and Biological Sciences, (DEB), Largo dell'Università, 01100 Viterbo, Italy; ^2^Complex Equipment Center, Tuscia University, Largo dell'Università, 01100 Viterbo, Italy

## Abstract

The omega-3 polyunsaturated fatty acids (*ω*-3 PUFAs) are a class of lipids that has been shown to have beneficial effects on some chronic degenerative diseases such as cardiovascular diseases, rheumatoid arthritis, inflammatory disorders, diabetes, and cancer. Among *ω*-3 polyunsaturated fatty acids (PUFAs), docosahexaenoic acid (DHA) has received particular attention for its antiproliferative, proapoptotic, antiangiogenetic, anti-invasion, and antimetastatic properties, even though the involved molecular mechanisms are not well understood. Recently, some *in vitro* studies showed that DHA promotes the inhibition of glycolytic enzymes and the Warburg phenotype. For example, it was shown that in breast cancer cell lines the modulation of bioenergetic functions is due to the capacity of DHA to activate the AMPK signalling and negatively regulate the HIF-1*α* functions. Taking into account these considerations, this review is focused on current knowledge concerning the role of DHA in interfering with cancer cell metabolism; this could be considered a further mechanism by which DHA inhibits cancer cell survival and progression.

## 1. Introduction

*ω*-3 polyunsaturated fatty acids (PUFAs), such as docosahexaenoic acid (DHA) and eicosapentaenoic acid (EPA), are essential fatty acids (FAs) that have beneficial effects on some chronic degenerative diseases such as cardiovascular diseases [1, 2], rheumatoid arthritis [3], diabetes [4], several autoimmune diseases [5, 6], and cancer [7, 8]. The EPA is a long-chain *ω*-3 PUFA that has 20 carbon atoms and 5 double bonds (20 : 5); DHA has a longer chain, 22 carbon atoms, and 6 double bounds (22 : 6). DHA and EPA, as well as the other FAs, once ingested, give a substantial contribution to the physical properties of biological membranes, including membrane organization, ion permeability, elasticity, and eicosanoid formation [9–11]. Taking into account these considerations, dietary DHA and EPA were established as significant nutrients involved in metabolic regulation. Moreover, some studies have established the capability of EPA, as well as in particular of DHA, to influence cancer proliferation [12], apoptosis [12, 13], and differentiation [12], as well as to inhibit angiogenesis [14], tumour cell invasion [15], and metastasis [16]. These data suggest that DHA can exert antitumour activity [17]. Despite the knowledge gained about the mechanisms associated with the anticancer effects of *ω*-3 PUFAs, still today, studies report new discoveries to clarify the complex system of the involved pathways.

Metabolic dysfunction is one of the emerging hallmarks of cancer: cancer cells show a shift in energy production that is abnormally dependent on aerobic glycolysis, and thus some of the key effectors of glycolysis (enzymes and transporters) can be considered as promising targets for the therapeutic intervention against cancer [18].

Recently, some studies have reported that DHA could act as metabolic modulator of several metabolic pathways in cancer cells [19–21].

This review focuses on the investigations on the potential use of DHA as modulator of some targets of aerobic glycolysis and Warburg effect.

## 2. Warburg Effect

Glycolysis is a catabolic pathway that converts a glucose molecule into two pyruvate molecules, and finally it yields 2 ATPs. In normal cells, pyruvate is oxidized to CO_2_ and H_2_O generating 36 ATPs in the mitochondrial oxidative phosphorylation pathway. When adequate oxygen supply is not available, normal cells use anaerobic glycolysis, because mitochondrial functions are suppressed in absence of oxygen. Under anaerobic condition, the conversion of pyruvate to lactic acid is favoured because this is the only mechanism available to regenerate NAD^+^, the coenzyme for glyceraldehyde-3-phosphate dehydrogenase. The conversion of glucose into lactate generates only a fraction of energy from glucose (2 moles of ATP/mole of glucose). Therefore, normal cells use this less efficient pathway, in terms of energy production, only under anaerobic conditions.

In contrast cancer cells, even under highly aerobic conditions, primarily derive energy from glucose via glycolysis to lactic acid, a property first observed by Otto Warburg [22]. Since then, this “aerobic glycolysis” is known as the “Warburg effect.” Because glycolysis is far less efficient for ATP production compared to mitochondrial oxidative phosphorylation, it is usually associated with marked increases in glucose uptake and consumption [23], a phenomenon clinically exploited to visualize cancer using the glucose similar 18-fluorodeoxyglucose by positron electron tomography [24]. The preference of cancers for aerobic glycolysis, over the more energy-efficient oxidative phosphorylation pathway, has many advantages for cancer. Warburg initially proposed that there was a defect within the mitochondria of tumour cells and they were unable to use oxygen to produce ATP. This hypothesis has been largely disproven, because the majority of cancers are able to revert back to oxidative phosphorylation when lactic acid generation is inhibited [25]. Further studies suggested that aerobic glycolysis has arisen as an adaptation to hypoxic conditions. Tumours commonly are located in an environment with fluctuating oxygen levels, alternating between normoxic and hypoxic conditions. The use of oxygen-independent glycolysis would confer a proliferative advantage to cancer cells, making them less susceptible to hypoxic stress during episodes of spontaneous hypoxia [26, 27]. However, this theory does not explain why these cells still undergo aerobic glycolysis when adequate oxygen amount is available. A more likely theory is that cancerous cells could favour aerobic glycolysis because of the large number of produced carbon-based intermediates, which may be useful in proliferative processes. Advantage of aerobic glycolysis lies in the incomplete utilization of glucose, allowing upstream intermediates to be redirected for biosynthesis, thereby providing cancer cells with an abundance of building blocks for synthesis of essential cellular components such as macromolecules. For example, glucose-6-phosphate, a metabolic intermediate of glycolysis, is used for nucleic acid synthesis through pentose phosphate pathway to support cell proliferation, as well as the large amount of pyruvate that is shunted from tricarboxylic acid cycle (TCA cycle) in mitochondria to lactate production through the upregulation of pyruvate kinase M2 isoform (PK-M2) and lactate dehydrogenase A (LDH-A) [18, 28, 29]. Another advantage of Warburg effect is the acidification of the microenvironment by lactic acid: aerobic glycolysis leads to an accelerated lactate secretion, which can acidify the surrounding extracellular matrix and facilitate angiogenesis and tumour metastasis [30]. In addition to the dependency on glycolysis, cancer cells exhibit other metabolic characteristics, such as increased fatty acid synthesis and glutamine metabolism. A pyruvate amount is utilized by a truncated trycarboxylic acid for lipid synthesis required for cell membrane formation during division, by exporting acetyl-CoA from the mitochondrial matrix to the cytoplasm. Enhanced fatty acid synthesis allows a quick tumour cell proliferation, conferring both a growth and survival advantage [31]. Glutamine is the most abundant amino acid in plasma and it constitutes an important additional energy source in tumour cells, especially when glycolytic energy production is low. The degradation products of glutamine (glutamate and aspartate) are necessary for rapidly proliferating cells by acting as aminoacid precursors [32]. Although the mechanisms underlying the Warburg's effect have not been completely understood, complex interactions between the major oncogenic pathways have been known to promote the glycolytic phenotype in cancer cells [33]. Since oncogenic activation is often thought as an early event in cancer development and progression, aerobic glycolysis could be a consequence of the oncogenic alteration.

### 2.1. Oncogenic Signalling and the Glycolytic Phenotype of Cancer Cells

The altered metabolic phenotype of cancer usually does not result from mutations in specific metabolic genes, except for rare mutations in two enzymes of the TCA, succinate dehydrogenase (SDH) and fumarate hydratase (FH), but rather is the result of mutations in metabolic regulators. A number of oncogenes, such as c-Myc, some tumour suppressors like p53, and hypoxia inducible factors-1 (HIF-1) have been linked to the dysregulation of glucose transport, TCA cycle, glutaminolysis, glycolysis, and hypoxic protection [34, 35]. Several genes are involved in hypoxic protection and their alteration results in an upregulation of glycolysis. Among them, the hypoxia-inducible transcription factors alpha (HIF-1*α*) is one of the most important factors involved in this mechanism. Indeed, under hypoxic conditions, HIF-1*α* becomes stabilized and forms a heterodimeric transcription complex with HIF-1*β*, which activates over 100 downstream genes important in hypoxic survival. Its targets include glycolytic enzymes (hexokinase, aldolase, and lactate dehydrogenase A), glucose transporters (GLUT family transporters), angiogenic factors (VEGF), haematopoietic factors (erythropoietin), and antiapoptotic factors (Bcl-2, IAP-2). In the presence of oxygen, HIF-1*α* activity is negatively regulated at post-translational level, by a family of oxygen-dependent prolyl asparagine hydroxylases (PHD), which begin the enzymatic sequence that leads to ubiquitination and proteolytic degradation, mediated by the Von Hippel-Lindau (VHL) protein [36]. In different types of tumours, even in conditions of normoxia, the HIF-1*α* protein levels are elevated, resulting from loss-of-function mutations targeting its negative regulator VHL tumour suppressor. Moreover, HIF-1*α* was found to be evoked in response to other stimuli, including radiation and reactive oxygen species (ROS), besides oncogenic signalling by ras, v-src, MEK-ERK, EGFR, and PI3K-AKT-mTOR pathways [37]. In particular, the PI3K-AKT-mTOR pathway plays a central role in growth factor signalling and glucose homeostasis. Indeed, mTORC1 (mammalian target of rapamycin complex 1), besides increasing HIF-1*α* protein by inducing its mRNA translation, promotes cell growth by regulating multiple biosynthetic processes, including ribosome biogenesis and protein and lipid synthesis. Two classes of direct downstream targets of mTORC1 are the ribosomal protein S6 kinases (S6K1 and S6K2) and the eukaryotic initiation factor 4E- (eIF4E-) binding proteins (4E-BP1 and 4E-BP2), both of which control specific steps in the initiation of cap-dependent translation [38, 39]. Aberrantly elevated mTORC1 activity detected in the majority of human cancers is mainly due to dysregulation of upstream signalling pathway. The serine/threonine kinase Akt was recognized as a major upstream activator of mTORC1; Akt inactivates the tuberous sclerosis complex (TSC) proteins, which is a negative regulator of mTORC1. Akt is regulated in turn by phosphatidylinositol 3-kinase (PI3K), which is a transducer of growth factor effects on cell survival [40, 41]. The Akt-activating ability of PI3K is opposed to the tumour suppressor PTEN (for phosphatase and tensin homolog deleted on chromosome 10), a phospholipid phosphatase that directly antagonized the PI3K activity [42]. Another way by which Akt activates mTORC1 is through the indirect inhibition of the AMP-kinase (AMPK), the central regulator of cell metabolism. Cells with hyperactive Akt accumulate high levels of ATP, which inactivate AMPK; instead elevated cytosolic levels of AMP activate AMPK, which phosphorylates and activates TSC. The activation of TSC by AMPK leads to inhibition of mTORC1 signalling [43]. AMP causes allosteric change that promotes the phosphorylation of Thr-172 in the activation loop of AMPK. This phosphorylation is necessary for the full activation of AMPK, which may be performed by the serine-threonine kinase LKB1 [44]. The AMPK phosphorylation is also mediated by the tumour suppressor p53. Besides AMPK, p53 has most well characterized targets which are involved in cell cycle regulation and apoptosis, but some studies have identified also several metabolic enzymes p53-regulated, like glucose transporter proteins 1–4 (GLUTs 1–4), hexokinase (HK), phosphofructokinase (PFK), and pyruvate dehydrogenase kinase (PDK) [45]. Therefore, the suppressive effects of AMPK signalling on glycolytic phenotype require the presence and the activation of tumour suppressive mechanisms mediated by p53, PTEN, TSC, and LKB1, but since they are frequently inactivated in cancers, the expression of the Warburg phenotype is promoted [37].

## 3. DHA as Modulator of the Metabolic Functions of Cancer Cells

Several biological mechanisms and pathways have been proposed to explain the health benefits of *ω*-3 PUFAs, including the ability to interact with energetic metabolism [46]. The metabolic changes induced in several tissues by *ω*-3 PUFAs have been long known. In particular EPA and DHA have been shown to act as hypolipidemic agents exerting prophylactic effects on cardiovascular diseases and improving insulin sensitivity [47, 48]. In liver *ω*-3 PUFAs inhibit the expression of genes encoding glycolytic and lipogenic enzymes both* in vivo* and* in vitro* [49, 50], and in white adipose tissues EPA and DHA regulate mitochondrial function, especially oxidative phosphorylation [51]. Since DHA and EPA are able to interfere with metabolic functions, it is tempting to affirm that these effects on cancer cell metabolism could be a further possible mechanism for inhibiting cancer survival and progression. This assumption is sustained by recent works, in which it has been demonstrated that *ω*-3 PUFAs are able to counteract the Warburg effect. In a proteomic, metabolomic, and interactomic integrated study, realized on human pancreatic PACA-44 cell line, it was found that DHA-induced apoptosis is preceded by a metabolic switch from glycolysis towards Kreb's cycle [19]. Indeed, in this paper proteomic and interactomic analysis identified several proteins related to overactivated oxidative metabolism in DHA treated cells. This result suggested that DHA causes an increase of energy production through mitochondrial pathway, resulting in the activation of aerobic metabolism. This was confirmed by metabolomic analysis in which metabolites related to glycolysis, like lactate and phosphoenol piruvate, decreased in DHA-treated cells, while metabolites involved in Kreb's cycle and pentose phosphate pathway, like *α*-ketoglutarate and NADPH, are significantly accumulated upon DHA-treatment. Moreover, from metabolomic analysis it was shown that glutathione/oxidized-glutathione (GSH/GSSG) ratio remained unaltered in DHA-supplemented cells in comparison to controls, despite the increase of oxidative stress in DHA-treated cells. The absence of the GSSG intracellular accumulation is explained by considering that the switch from glycolytic pathway towards pentose phosphate pathway leads to the accumulation of NADPH, essential coenzyme in the reduction processes of several antioxidant enzymes, and biomolecules, like GSH. For the first time, this proteomic and metabolomic study highlights the DHA ability to modulate glycolytic metabolism that might represent a further mechanism of growth inhibition and apoptosis activation established by *ω*-3 PUFAs treatment.

**Figure 1:**
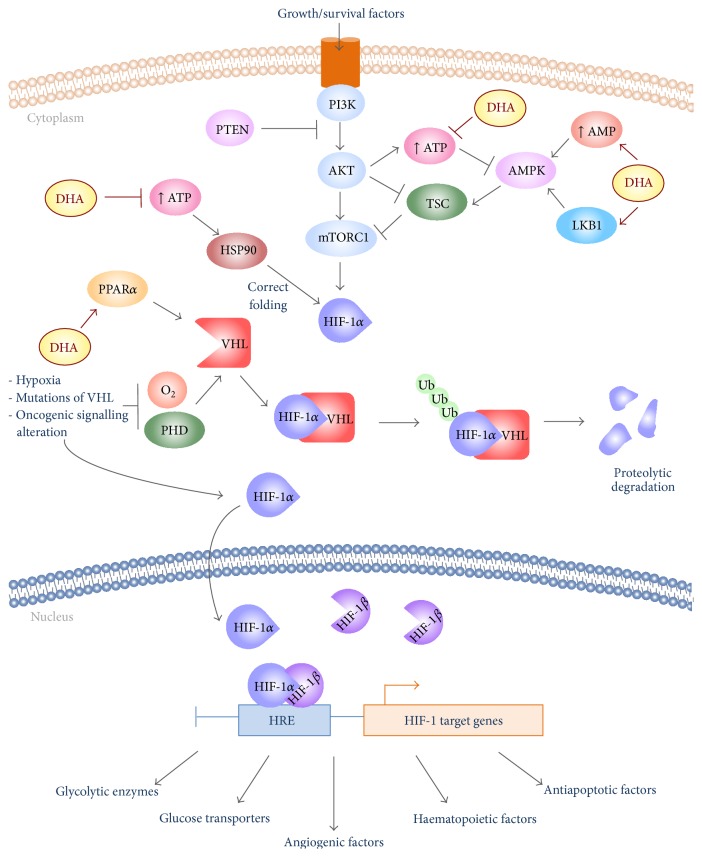
Schematic illustration of the mechanism by which DHA may interfere with the molecular signalling by activating glycolytic phenotype. The PI3K-Akt-mTORC1 pathway promotes the glycolytic phenotype, principally activating the transcription factor HIF-1*α*. HIF-1*α* is activated also by hypoxia, as well as by mutations of its regulator VHL. The accumulation of HIF-1*α* in the cytosol determines its heterodimerization with the subunit HIF-1*β*, forming the active HIF-1 complex. HIF-1 upregulates a wide network of genes by binding to hypoxia response elements (HRE). DHA interferes at various sites of this pathway, and then it is able to attenuate bioenergetic function and Warburg metabolism. DHA treatment increases the LKB1 protein expression and AMP cytosolic levels, necessary events to activate the AMPK pathway. Active AMPK inhibits mTORC1 signalling, via phosphorylation of TSC protein. Moreover, DHA alters cancer cell metabolism by interfering with the processes implicated in the stabilization of HIF-1*α*. Indeed, the reduction of cytosolic ATP levels induced by DHA prevents the proper functioning of HSP90, molecular chaperon necessary for folding of HIF-1*α*. Moreover, DHA destabilizes HIF-1*α* promoting its proteolytic degradation via PPAR*α* activation.

In a recent work, it was shown that DHA decreases the bioenergetic functions and metabolic reprogramming of breast cancer cell lines [20]. In this study, two metabolically distinct breast cancer cell lines were utilized, BT-474 and MDA-MB-231, representing mitochondrial and glycolytic phenotypes, respectively, and nontumorigenic breast epithelial cell line, MCF-10A, to identify the efficacy of DHA in multiple metabolic pathway. The extracellular acidification rate (ECAR), representative of glycolysis, and the oxygen consumption rate (OCR), representative of oxidative phosphorylation, were analysed in response to DHA treatment. Both parameters significantly decreased in the two cancer cell lines in a dose-dependent manner in response to DHA supplementation, compared with untreated cells but not in nontumorigenic control. These findings suggest that, independently of metabolic phenotype of cancer cells, DHA is able to change the bioenergetic profile. Moreover, DHA selectively targets malignant cell lines, since no effect was observed in the MCF-10A nontransformed cell line. The authors argue that the ability of DHA to interfere, not only with the glycolytic activity, but also with the mitochondrial respiration, is due to its capacity to alter the mitochondrial structure and function. Indeed, from the literature it is known that DHA may modify the mitochondrial phospholipid composition and alter the activity of essential inner membrane proteins and channels; this could lead to a reduction of mitochondrial bioenergetic function [52]. The reduction of oxidative phosphorylation is an effect that counteracts with the results obtained in the above discussed work of D'Alessandro et al., where the DHA-treated pancreatic cancer cell line showed a shift from glycolysis to Kreb's cycle [19]. It is possible that the DHA effects on mitochondrial functions are different among cell types. This may depend on the functional state of mitochondria themselves, as demonstrated in work of Suchorolski et al. [53]. In this work, it was compared with ECAR and OCR in four cell lines derived from Barrett's oesophagus (BE), a premalignant condition associated with an increased risk of oesophageal adenocarcinoma (EA), in response to metabolic inhibitors. The treatment with 2-deoxyglucose (2-DG), a competitive inhibitor of glycolytic pathway, increases the OCR value, only in the cell line CP-D. From the analysis of nuclear and mitochondria genome it was found that the CP-D line had the fewest number of mitochondrial genome mutations, among all cell lines. Since this cell line has functional mitochondria, it is able to revert the glycolytic metabolism towards oxidative phosphorylation [53]. Moreover, it is possible that the increased activity of Kreb's cycle, as a result of glycolysis inhibition, may be associated with the ability of some cells to oxidize alternative substrates like glutamine or fatty acids, which provide TCA cycle metabolites [54]. In the work of Mouradian et al., it was shown that the decrease of bioenergetic functions is associated with the reduction of HIF-1*α* expression and activity in DHA-treated breast cancer cell lines [20] (Figure 1). Further investigation found a reduction of downstream transcriptional targets of HIF-1*α*, glucose transporter 1 (GLUT1), and lactate dehydrogenase (LDH). The authors hypothesize that the DHA-induced decrease of HIF-1*α* can occur by two modalities: the first hypothesis expected that DHA induces degradation of HIF-1*α* protein through activation of PPAR*α*. This consideration comes primarily from extensive scientific evidences that showed the ability of DHA and its metabolites to activate peroxisome proliferator-activated receptors (PPARs) [55, 56]. Moreover, in a recent work it has been demonstrated that the activation of PPAR*α* by clofibrate suppressed HIF-1*α* signalling by increasing degradation of HIF-1*α*. The activated PPAR*α* would seem to increase the interaction of HIF-1*α* with VHL, which enhances the ubiquitin-proteasome degradation pathway [57]. The other hypothesized mechanisms provide that the decrease of HIF-1*α* is due to a dysfunction of the HSP90 complex, which is required for a correct folding of this transcription factor [58]. Decreases of intracellular ATP levels attenuate the function of the HSP90 molecular chaperone; DHA treatment determines a reduction of ATP and so the disruption of the HSP90 function (Figure 1). The metabolic stress induced by DHA is demonstrated also by an increase in phospho-Thr172-AMPK in treated cells. This result is important evidence that DHA is able to modulate the AMPK pathway, which is implicated in reducing cell proliferation and in regulation of cell metabolism.

In another work, a further mechanism has been proposed by which DHA is able to regulate the AMPK signalling [21]. Indeed, in this paper it was shown that DHA enhanced the tumour suppressor function of LKB1 in breast cancer cell lines. AMPK is a direct target of LKB1, and, by activating LKB1 signalling, DHA treatment leads to phosphorylation and activation of AMPK (Figure 1). The results have shown that pAMPK, in turn, suppresses the mTORC1 signalling and the relative downstream targets, S6K and eIF4E. The suppression of mTORC1 decreased the capacity of cells to execute glycolysis; in fact, the expression of glycolytic enzymes, like hexokinase 2 and lactate dehydrogenase, was decreased in presence of DHA. Consequently, with the decrease of these enzymes a reduction of lactate production has occurred and then of extracellular acidification. These events lead to a decrease in the migration potential of the cells, as demonstrated in this study by migration assay on breast cancer cell lines treated with DHA. The DHA-induced reduction of the lactate concentration may be considered another potential mechanism by which DHA exerts the anti-invasive capacity [17].

## 4. Conclusions

Based on the results obtained from these studies, it can be stated that DHA interferes with the glycolytic phenotype of cancer cells. However, the bioenergetic dysfunction and the metabolic reprogramming induced by DHA differ among cell lines. Indeed, in pancreatic PACA-44 cell line, the DHA-induced glycolysis depression is followed by Kreb's cycle activation; instead, in some breast cancer cell lines DHA treatment leads to the inhibition of both glycolytic and mitochondrial activity. The decrease of the bioenergetic functions in breast cancer seems to be due to the ability of DHA to activate the AMPK protein, decreasing the ATP levels and activating the LKB1 protein. Probably these mechanisms are connected and contribute to the negative regulation of HIF1-*α*, because both reduction of ATP and inhibition of mTOR signalling lead to suppression of this transcription factor. Further studies are needed to demonstrate whether DHA is able to interfere with cell metabolism in other types of cancer and if other mechanisms are involved. Nevertheless, the studies reported here show that alteration of cell metabolism may be considered as a further mechanism by which DHA can contribute to impair cancer cell growth and survival and so this provides a new innovative strategy for cancer therapy through targeting cancer cell metabolism.
